# Potential Anti-obesogenic Effects of *Ginkgo biloba* Observed in Epididymal White Adipose Tissue of Obese Rats

**DOI:** 10.3389/fendo.2019.00284

**Published:** 2019-05-10

**Authors:** Bruna K. S. Hirata, Maysa M. Cruz, Roberta D. C. C. de Sá, Talita S. M. Farias, Meira M. F. Machado, Allain A. Bueno, Maria Isabel C. Alonso-Vale, Monica M. Telles

**Affiliations:** ^1^Department of Biological Sciences, Institute of Environmental, Chemical and Pharmaceutical Sciences, Universidade Federal de São Paulo, Diadema, Brazil; ^2^Department of Biological Sciences, College of Health, Life and Environmental Sciences, University of Worcester, Worcester, United Kingdom

**Keywords:** lipogenesis, obesity, *Ginkgo biloba* extract, high fat diet, fatty acid synthase

## Abstract

Exacerbated expansion of adipose tissue seen in diet-induced obesity leads to endocrine dysfunction and disturbance in adipokine secretion, with such abnormal profile positively associated with type 2 diabetes and other mild chronic inflammatory conditions. *Ginkgo biloba* extract (GbE), a mixture of polyphenols with antioxidant properties, has been recently investigated in a variety of experimental models of endocrine dysfunction, with several potentially beneficial effects identified, including improvement in insulin sensitivity in obese rats, and reduction of weight gain in ovariectomy-induced obesity and diet-induced obesity. The aim of this study was to investigate in high fat diet-induced obese male rats the effects of GbE supplementation for 2 weeks on adipocyte volume and adipose tissue lipid accumulation. GbE supplementation was effective in reducing energy intake in obese rats compared to the saline-treated placebo group. Epididymal adipocyte volume was reduced in GbE-supplemented rats, as were epididymal [1-^14^C]-acetate incorporation into fatty acids, perilipin (*Plin 1*) and fatty acid synthase (*Fasn*) mRNA, and FAS protein levels. Adipocyte hypertrophy in obesity is associated with insulin resistance, and in the present study we observed a reduction in the adipocyte volume of GbE-supplemented obese rats to dimensions equivalent to adipocytes from non-obese rats. GbE supplementation significantly reduced acetate accumulation and tended to reduce [^3^H]-oleate incorporation, into epididymal adipose tissue, suggesting a potentially anti-obesogenic effect in longer term therapies. Further studies that investigate the effects of GbE supplementation in other experimental models are required to fully elucidate its suggested beneficial effects on mild chronic inflammatory conditions.

## Introduction

The definition of white adipose tissue (WAT) as an inert mass for energy storage is long gone; over the last two decades the adipose tissue has been recognized as a dynamic tissue and key player in the modulation of energy metabolism (1, 2). Adipokines such as leptin, adiponectin, and tumour necrosis factor-α (TNF-α) have a direct effect on energy homeostasis and modulation of low-grade inflammation (3). The intake of high fat diets has the potential to not only disturb normal adipokine secretion but also to remodel adipose tissue by increasing adipocyte size and/or number, contributing to the development of a pro-inflammatory microenvironment (4, 5). These perturbations have been positively associated with metabolic disorders such as obesity, type 2 diabetes, non-alcoholic fatty liver disease (NAFLD), insulin resistance, and cardiovascular diseases (6, 7).

In obesity, particularly visceral obesity, enlarged WAT visceral adipocytes show dysregulated lipolysis, inducing high levels of circulating non-esterified fatty acids (NEFAs) (8, 9). NEFAs in normal circumstances are utilized as energy by tissues such as liver and muscle; however, when in excess they contribute to the development of insulin resistance (4, 9–11). Furthermore, in response to overnutrition, hypertrophic adipocytes contribute to increased circulating triacylglycerol (TAG) levels mainly from *de novo* lipogenesis, in which fatty acids (FA) are synthetized from non-lipid substrates, particularly carbohydrates, or from FA obtained from *ex-situ* lipid sources such as chylomicrons and very-low-density lipoproteins (VLDL) (12, 13). Visceral obesity seems to play a central role in the development of metabolic disorders, being associated with low-grade chronic inflammation and the production of pro-inflammatory cytokines which have the potential to trigger insulin resistance and endothelial dysfunction (14–16).

In this context, several pharmacological approaches have been tried for the treatment of obesity. However, more often than not such approaches were followed by undesired side effects, including psychiatric manifestations, increased risk of cardiovascular events, and others (17). Considering the dramatic increase in the prevalence of obesity over the last decades globally, a range of anti-obesogenic alternative supplementation therapies based on plant extracts (18) have been investigated.

More recently, *Ginkgo biloba* Extract (GbE) has been investigated as an alternative therapy for metabolic disorders associated with obesity. GbE, a herbal extract containing flavonoids, terpenoids, and terpene lactones (19), is a well-known phytotherapic compound often employed as coadjuvant supplement in neurodegenerative diseases (20, 21), NAFLD (22, 23), type 1 and 2 diabetes (24, 25). Previous findings from our research group showed that diet-induced obese (DIO) rats supplemented with GbE showed reduced food and energy intake, reduced body adiposity, improved insulin signalling and sensitivity, enhanced insulin receptor and AKT phosphorylation, and reduced NFκB-p65 phosphorylation in retroperitoneal adipose tissue (26, 27).

GbE may have a potentially therapeutic use for menopause-associated obesity; supplementation with 500 mg/kg of GbE stimulated hypothalamic serotonergic activity in ovariectomized rats (28). GbE isolated bioactive compounds have been demonstrated to stimulate lipolysis in 3T3-L1 adipocytes (29), and to inhibit adipogenesis through activation of the AMPK pathway (30). However, the effects of GbE supplementation on metabolic processes of visceral adipose tissue in DIO rats remain largely unknown. In view of the considerations highlighted above, the aim of the present study was to investigate the effects of GbE supplementation as a potentially anti-obesogenic effector for improvement in lipid metabolism of epididymal adipose tissue of DIO rats.

## Materials and Methods

### Ethical approval

This study was carried out in strict accordance with the recommendations of the Guide for the Care and Use of Laboratory Animals. The Committee on Animal Research Ethics of the Universidade Federal de São Paulo approved all procedures for the care of the animals used in this study (Process number: 8700110814).

### Animal Care

Two months-old male Wistar rats from Multidisciplinary Center for Biological Investigation in Laboratory Animals Science (CEMIB - Campinas, Brazil) were housed at 4 or 5 rats per cage and maintained in controlled lighting (12:12-h light/dark, lights on at 6:00 a.m.) and temperature (23°C ± 1°C) conditions, with *ad libitum* access to food and water.

Briefly, the high fat diet was prepared by mixing 40% (w/w) ground standard chow (Nuvilab®, Brazil, 2.7 kcal/g), with 28% (w/w) melted lard, 20% (w/w) casein powder, 10% (w/w) sucrose, 2% (w/w) soybean oil, and 0.02% (w/w) butylated hydroxytoluene (5.0 kcal/g). All the ingredients were thoroughly mixed and lukewarm drinking water added to obtain the consistency necessary to allow perfect homogenization of the mixture and production of pellets, which were subsequently dried in a forced ventilation oven at 60°C for 24 h. This diet provides 19.5% of energy from carbohydrate, 23.2% from protein, and 57.3% from fat, and has been demonstrated in previous studies to induce obesity (26, 27). The diet macronutrient composition was analysed in the laboratory of Bromatology and Microbiology of Foods, Universidade Federal de Sao Paulo, and the diet fatty acid composition was determined by gas chromatography (Table 1).

**Table 1:** Macronutrients and fatty acid composition (% of total fatty acids) of standard chow and high-fat diet.

	**Standard chow**	**High-fat diet**
Humidity (%)	9.1	1.1
Lipids (%)	4.5	31.6
Protein (%)	22.7	27.0
Carbohydrates (%)	35.9	27.5
Total fibre (%)	18.9	8.6
Fixed mineral residue (%)	8.9	4.2
Sodium chloride (%)	0.6	0.2
Calculated energy (kcal/g)	2.7	5.0
Fatty acid composition (% total)		
**SATURATED FATTY ACIDS**		
Myristic acid - C14:0	0.0	1.1
Palmitic acid - C16:0	14.6	21.4
Stearic acid - C18:0	3.6	11.6
Monounsaturated fatty acid		
Palmitoleic acid - C16:1n7	0.0	1.7
Vaccenic acid - C18:1n7	0.9	2.26
Oleic acid - C18:1n9	24.1	35.5
Eicosanoic acid - C20:1n9	0.3	0.6
**POLYUNSATURATED FATTY ACIDS**		
Linoleic acid - C18:2n6	55.4	21.4
Linolenic acid - C18:3n3	4.7	1.3

Over the course of 2 months for the development of obesity, normal fat diet-fed rats (NFD, *n* = 20) received the standard chow, while the high fat diet-fed rats (HFD, *n* = 54) received the lard enriched diet, as described above. All rats were weighted once weekly, and the food/energy intake was calculated by the difference between the food left from the food offered 24 h before. Food efficiency was calculated by the ratio of body weight gain (g) to food ingestion (g) weekly.

### GbE Supplementation

At the end of the 2 months induction period, the HFD group was randomly sorted into two subsets. The NFD group and the first HFD subset received 1 mL 0.9% saline by gavage daily for 2 weeks, whilst the second HFD subset (HFD+GbE) was orally gavaged with GbE 500 mg/kg, as previously described (31).

GbE was obtained from Huacheng Biotech Inc. (China), and the major bioactive compounds were flavone glycosides (25.21%), terpenoids (6.62%), ginkgolides A, B, C (3.09%), and bilobalides (2.73%). Food and energy intake were registered daily for the 2 weeks of supplementation. Body weight gain was calculated by the difference between the first and the last day of the 2 weeks period.

### Adipocyte Isolation

Adipocyte isolation was performed as previously described and optimized (32–34). Briefly, epididymal fat pads were diced in small fragments in a flask containing 4 mL of DMEM supplemented with HEPES (20 mM), glucose (5 mM), bovine serum albumin (BSA, 1%), and collagenase type II (1 mg/mL), pH 7.4 and incubated for ~40 min at 37°C in an orbital shaker. Isolated adipocytes were filtered through a plastic sieve (150 μm) and washed three times in fresh buffer without collagenase. After washing and brief spin, the medium was thoroughly aspirated, and the adipocytes harvested. Adipocytes were photographed under an optic microscope (× 100 magnification) coupled to a microscope camera (AxioCam ERc5s; Zeiss®, Oberkochen, Germany). Mean adipocyte volume (4/3 x π x r^3^), expressed in pL, was calculated by averaging the measurement of 50 cells, employing AxioVision LE64 software.

The percentage of adipocytes contained in the total cell suspension was determined using 40 μL of the cell suspension in EHB buffer placed in glass capillary and subjected to centrifugation (500 g for 1 min). The total volume of the suspension corresponds to 100% and the volume of adipocytes obtained after centrifugation gives the percentage of adipocytes of the sample.

### Lipolysis

Lipolysis was estimated as concentration of glycerol release into the incubation medium. For this, isolated epididymal adipocytes (1 × 10^6^ cells/mL) were incubated in Krebs/Ringer/phosphate buffer (pH 7.4) containing BSA (20 mM) and glucose (5 mM) for 30 min at 37°C in the presence or absence of isoproterenol (2 × 10^−6^ M). The reaction was stopped by cooling the flasks on ice, and the media was carefully collected for measurement of glycerol release employing a free glycerol determination kit (Sigma®). Results are expressed as nmol of glycerol / 1 × 10^6^ adipocytes.

### Fatty Acid Uptake

Fatty acid uptake, measured by intracellular accumulation of [^3^H]-oleate, was quantified following previously established methods (32, 33). Briefly, isolated epididymal adipocytes (1 × 10^6^ cells/ mL) were incubated in Krebs/Ringer/phosphate buffer (pH 7.4) containing BSA (1%), glucose (2 mM), [^3^H]- oleate (100 μM, 1850 Bq/tube or well) for 2 h at 37°C in a water bath. At the end of the incubation period, the mixture was transferred to 1.5 mL tubes containing 400 μL of silicone oil and centrifuged for 30 s. The cell pellet on top of the oil layer was transferred to polypropylene tubes containing 2.5 mL of Dole's reagent for lipid extraction. After addition of *n*-heptane (1.5 mL) and distilled water (1.5 mL), tubes were vortexed and the mixture decanted for 5 min. An aliquot of the upper phase was collected into a scintillation vial for the determination of radioactivity incorporated into TAG (1450 LSC, Couter MicroBeta, Trilux; Perkin Elmer, Waltham®, MA, USA). Results are expressed as nmol of oleate/1 × 10^6^ cells/h.

### Incorporation of [1-^14^C]-Acetate Into Triacylglycerol

*De novo* lipogenesis in epididymal adipocytes was estimated by incubating isolated cells (1 × 10^6^ cells/mL) in Krebs/Ringer/phosphate buffer (pH 7.4) containing BSA (1%), glucose (2 mM), and [1-^14^C]-acetate (1 mM, 1850 Bq/tube or well) for 2 h at 37°C in a water bath and processed as described previously (32, 33). Results are expressed as nmol of acetate incorporated into TAG/1 × 10^6^ cells/h.

### RNA Extraction and Quantitative Real-Time Polymerase Chain Reaction (qPCR)

Total RNA from epididymal adipocytes was extracted with Trizol®reagent (Invitrogen Life Technologies®) and reversely transcribed into cDNA using a High-Capacity cDNA kit (Applied Biosystems®) following the manufacturer's instructions. Gene expression was evaluated by real-time qPCR using a Rotor Gene (Qiagen®) and SYBR Green as fluorescent dye with Beta2M as housekeeping gene.

Primers used and annealing temperatures were employed as followed: FAS (*Fasn*) (5′-3′ sense: GAGTCCGAGTCTGTCTCCCGCTTGA; 5′-3′ antisense: GCCGTGAGGTTGCTGTTGTCTGTAG; 64°C; NM_017332); HSL-(*Lipe*) (5′-3′ sense: CCTGCTGACCATCAACCGAC; 5′-3′ antisense: CCTCGATCTCCGTGATATTCCAGA; 60°C; NM_012859); Perilipin 1 (*Plin1*) (5′-3′ sense: CCTCTTGCCCCGATCTGGAT; 5′-3′ antisense: CAAGCCCCAAGGATGCCTTA; 60°C; NM_013094); beta-2 microglobulin (Beta2M) (5′-3′ sense: CTC AGT TCC ACC CAC CTC AG; 5′-3′ antisense: GCA AGC ATA TAC ATC GGT CTC G; 56°C; NM_012512). The 2^−ΔΔ*Ct*^ method (35) was used to evaluate the relative quantification of amplification products relative to the control group NFD. At least one sample from each group was included in each run, and reactions were carried out in triplicate.

### Protein Expression Estimation by Western Blotting

Epididymal fat pads were removed and immediately homogenized in 1 mL lysis buffer (100 mM Tris, pH 7.5, 10 mM EDTA, 0.1 mg/mL aprotinin; 2 mM PMSF; 10 mM sodium orthovanadate; 100 mM sodium fluoride; 10 mM sodium pyrophosphate; and 10% TritonX-100), homogenized and centrifuged at 16,000 g for 40 min at 4°C.

Fifty micrograms of protein were separated in 10% SDS-PAGE, transferred onto nitrocellulose membranes and incubated with primary antibody anti-Fatty Acid Synthase (FAS - Santa Cruz®– SC20140), anti-phospho-hormone-sensitive lipase (p-HSL Cell Signaling® - #4139) and anti-hormone-sensitive lipase (HSL Cell Signaling®- #4107). Membranes were subsequently incubated with peroxidase-conjugated antibody (Cell Signaling®- #7074) followed by chemiluminescence detection. β-tubulin (Cell Signaling®- #2146) levels were used as internal standard. Quantitative analysis was performed with ImageJ software (ImageJ®, version 2.0, Maryland, USA). In all experiments, at least one sample from each group was analysed simultaneously and the results were expressed as percentage change relative to NFD group levels, as described previously (27). Representative pictures of Western blotting gels are provided in the Supplementary Data Sheet 1.

### Statistical Analysis

Data were subjected to quality tests such as Shapiro–Wilk (normality) and/or Levenne (homogeneity). If necessary, data were standardized according to the log transformation. Descriptive analysis was performed using mean ± SEM, and measurements were taken from distinct samples. Comparisons of body mass, food intake, body weight gain, and food efficiency between NFD and HFD were assessed by *Student's t-test* for independent samples. One-way ANOVA followed by Tukey *post-hoc* test was employed to assess the effects of supplementation among NFD, HFD, and HFD+GbE. Mann-Whitney test was performed to analyse acetate incorporation into lipids, since this was a non-linear parameter compared between HFD and HFD+GbE groups only. Statistical analysis was performed using SPSS®software version 20. The number of samples used in each experiment is shown in the respective Results section. The level of significance adopted was set at *p* ≤ 0.05.

## Results

### Food, Energy Intake, and Body Weight Gain During the Obesity Induction Period

**Figure 1:**
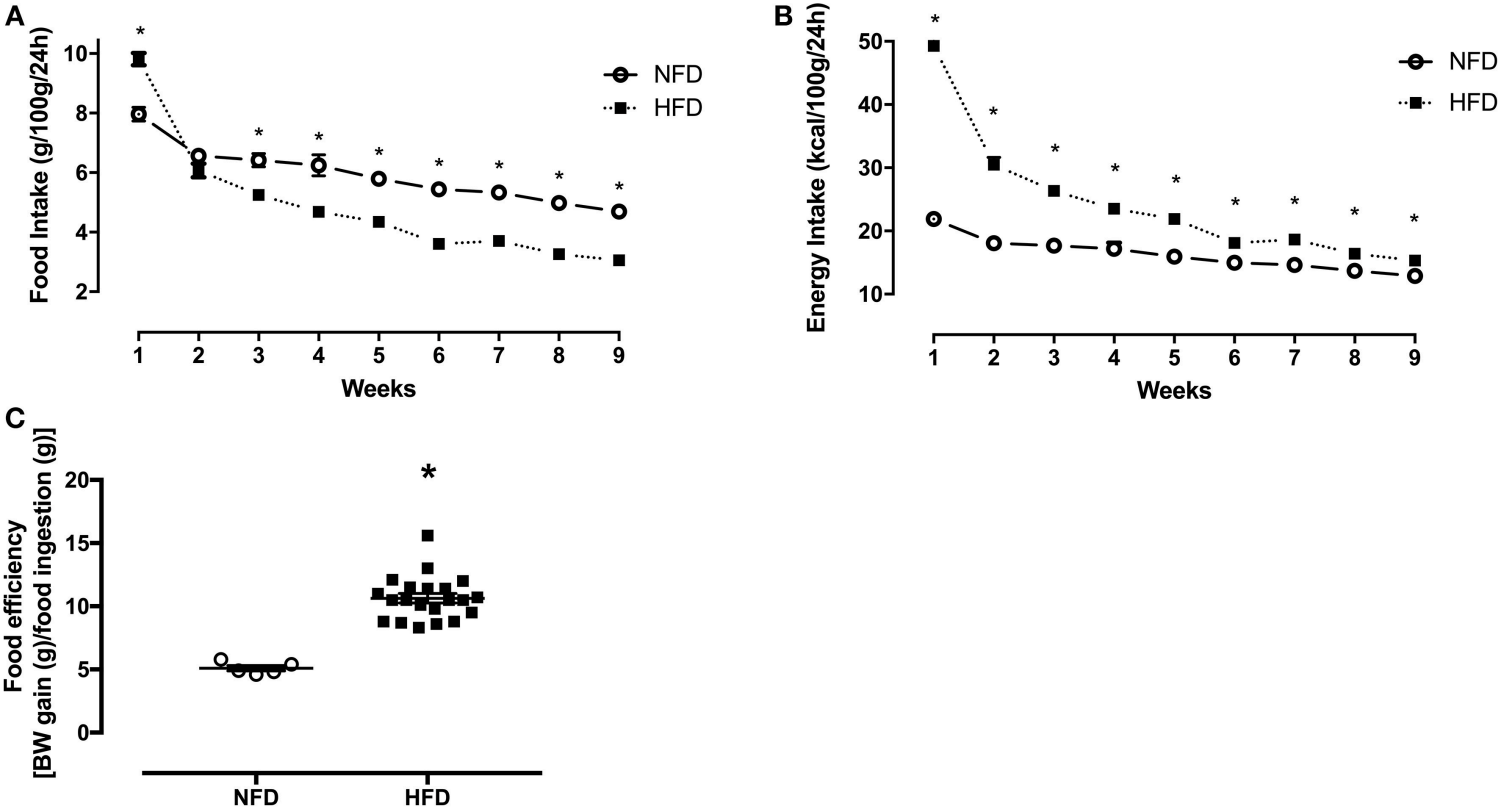
Food intake, energy intake, and food efficiency. Food intake (g/100 g/24 h) **(A)**, energy intake (kcal/100 g/24 h) **(B)**, and food efficiency [BW gain (g)/food ingestion(g)] **(C)** of normal-fat diet (NFD; *n* = 5) and high-fat diet (HFD; *n* = 13) groups during the obesity induction period. ^*^p < 0.05 vs. NFD (Student's *t*-test).

Food intake (g/100 g/24 h) was ~23% higher (*p* < 0.001) in the HFD group in the first week of feeding (Figure 1A). From the third week onwards, HFD rats ingested ~29% (*p* = 0.001) less food in grams, but their energy intake (kcal/100 g/24 h) remained ~44% (*p* = 0.001) higher than NFD in the same period (Figure 1B). Additionally, food efficiency [BW gain (g) / food ingestion (g)] was significantly higher in HFD (10.64 ± 0.37) than in NFD (5.09 ± 0.21) (*p* < 0.001) (Figure 1C).

**Figure 2:**
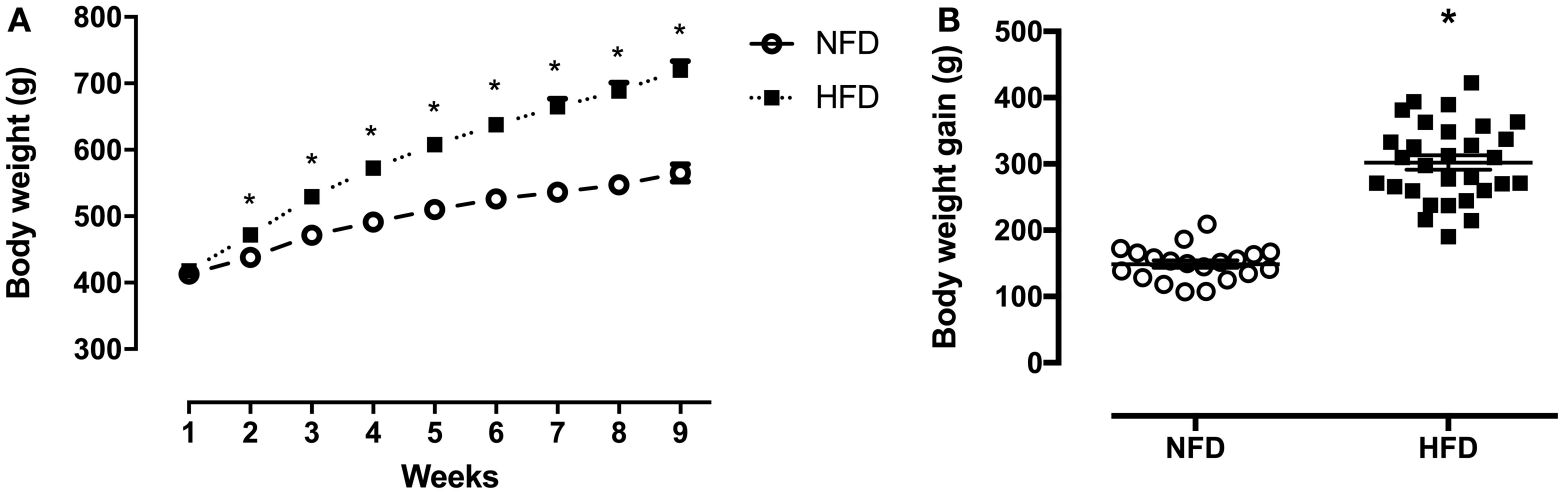
Body composition. Body weight (g) during the obesity induction period **(A)** and body weight gain (difference between initial and final BW) (g) **(B)** of normal-fat diet (NFD; *n* = 20) and high-fat diet (HFD; *n* = 29) ^*^p < 0.05 vs. NFD (Student's *t*-test).

Throughout the 2 months of obesity-induction period, both groups showed continued body weight increase. From weeks 2 to 9, the HFD group gained around 16% (*p* < 0.001) more weight than the NFD group (Figure 2A). The high-fat diet intake resulted in an extra 153.3 g of body weight gain in relation to the chow-fed rats (*p* < 0.001) (Figure 2B).

### Food, Energy Intake, and Body Weight Gain During the GbE Supplementation Period

**Figure 3:**
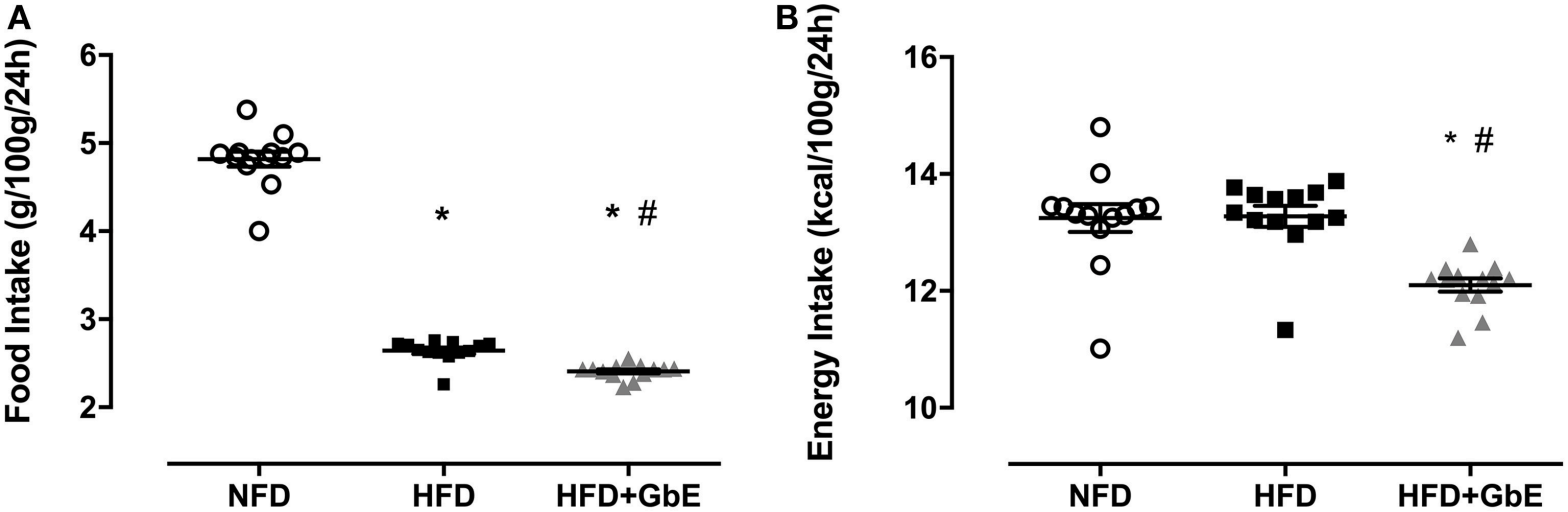
Accumulated food and energy intake after supplementation. Accumulated food intake (g/100 g/24 h) **(A)** and energy intake (kcal/100 g/24 h) **(B)** of normal-fat diet (NFD; *n* = 10), high-fat diet (HFD; *n* = 24) and high-fat diet plus GbE supplementation (HFD+GbE; *n* = 24). ^*^p < 0.05 vs. NFD and #*p* < 0.05 vs. HFD (One-way ANOVA).

**Figure 4:**
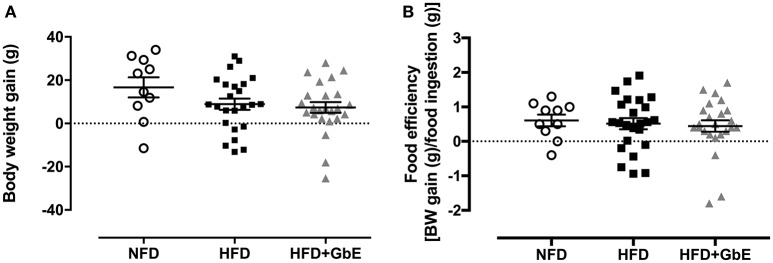
Body composition after supplementation. Body weight gain (g) **(A)** and food efficiency [BW gain (g)/food ingestion(g)] **(B)** of normal-fat diet (NFD; *n* = 10), high-fat diet (HFD; *n* = 24) and high-fat diet plus GbE supplementation (HFD+GbE; *n* = 24). ^*^p < 0.05 vs. NFD (One-way ANOVA).

At the end of the 2 months obesity-induction period (NFD vs. HFD), the HFD group was randomly sorted into two subsets: HFD (*n* = 24) or HFD+GbE (*n* = 27). For the two following weeks, both HFD and HFD+GbE groups showed reduced food intake in comparison to NFD (45.1 and 50.1%, respectively; *p* < 0.001), but the HFD+GbE group showed reduced food intake in comparison to HFD (8.9%, *p* = 0.014). The energy intake was statistically similar between NFD and HFD; however, the HFD+GbE energy intake was significantly lower than NFD (8.7%, *p* = 0.001) and HFD (8.9%, *p* < 0.001) (Figures 3A,B). Body weight gain (g) and food efficiency [BW gain (g)/ food ingestion (g)] during the 2 weeks supplementation period were monitored; however, no statistically significant differences were found (Figures 4A,B).

### Adipocyte Volume and Metabolism

**Figure 5:**
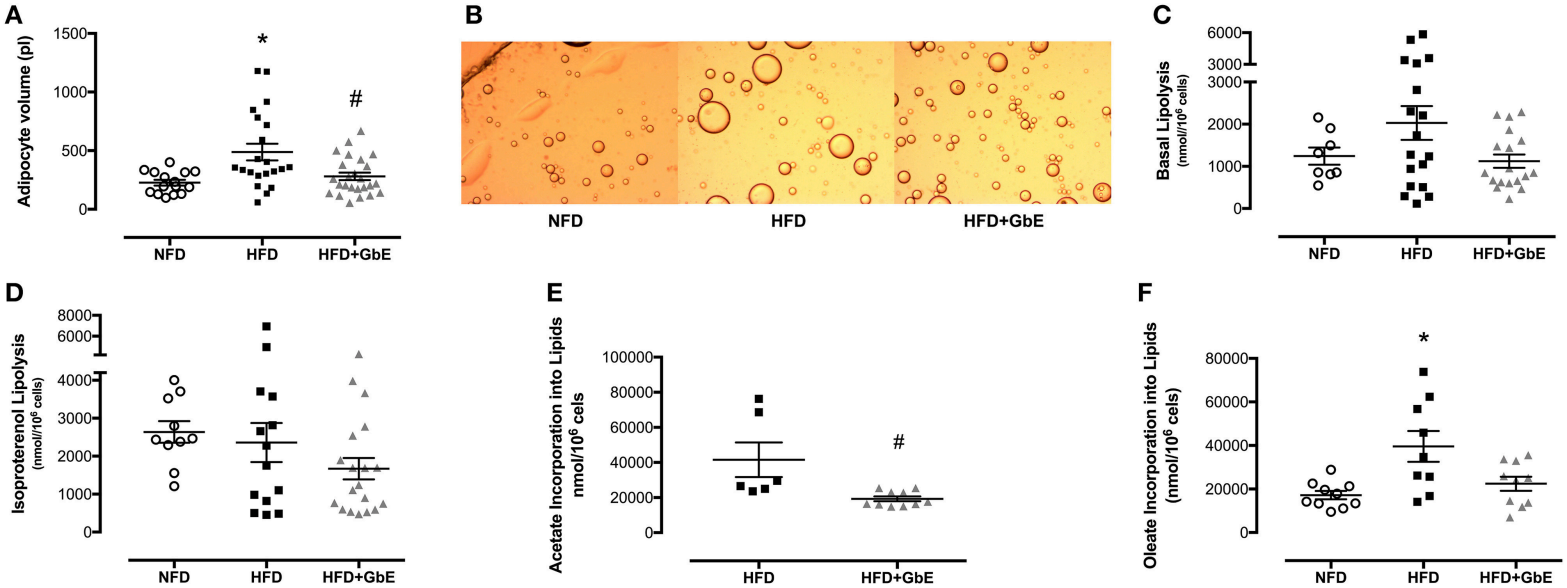
Adipocyte metabolism. Adipocyte volume (pL) **(A)**, photomicrograph **(B)**, basal lipolysis (nmol/10^6^cells) **(C)**, isoproterenol-stimulated lipolysis (nmol/10^6^ cells) **(D)**, oleate incorporation into lipids (nmol/10^6^ cells) **(E)**, and acetate incorporation into lipids **(F)** (nmol/10^6^ cells) of normal-fat diet (NFD; *n* = 16–6), high-fat diet (HFD; *n* = 22–9), and high-fat diet plus GbE supplementation (HFD+GbE; *n* = 26–10) groups. ^*^p < 0.05 vs. NFD and #*p* < 0.05 vs. HFD (One-way ANOVA and Mann-Whitney test on acetate incorporation).

Adipocyte volume was significantly larger in HFD than NFD (114%, *p* = 0.01), but GbE supplementation reduced this parameter by 42.5%, as compared to HFD (*p* = 0.03). Adipocyte volume was statistically similar between HFD+GbE and NFD (Figures 5A,B).

No statistically significant differences were found in lipolysis rate (nmol/10^6^ cells) or isoproterenol-induced lipolysis rate (Figures 5C,D) amongst the three groups. However, oleate incorporation into lipids was 130% higher (*p* = 0.01) in HFD (39573.1 nmol/10^6^ cells) than NFD (17189.9 nmol/10^6^ cells). Additionally, GbE supplementation showed a strong tendency to reduce this parameter by 43% (*p* = 0.06) (22412.7 nmol/10^6^ cells) in relation to HFD (Figure 5E). Acetate incorporation into lipids (nmol/10^6^ cells) was reduced by 115% (*p* = 0.003) in HFD+GbE (19286.5 nmol/10^6^ cells) in relation to HFD (41560.2 nmol/10^6^ cells) (Figure 5F).

### Epididymal Adipose Tissue mRNA Expression and Protein Synthesis

**Figure 6:**
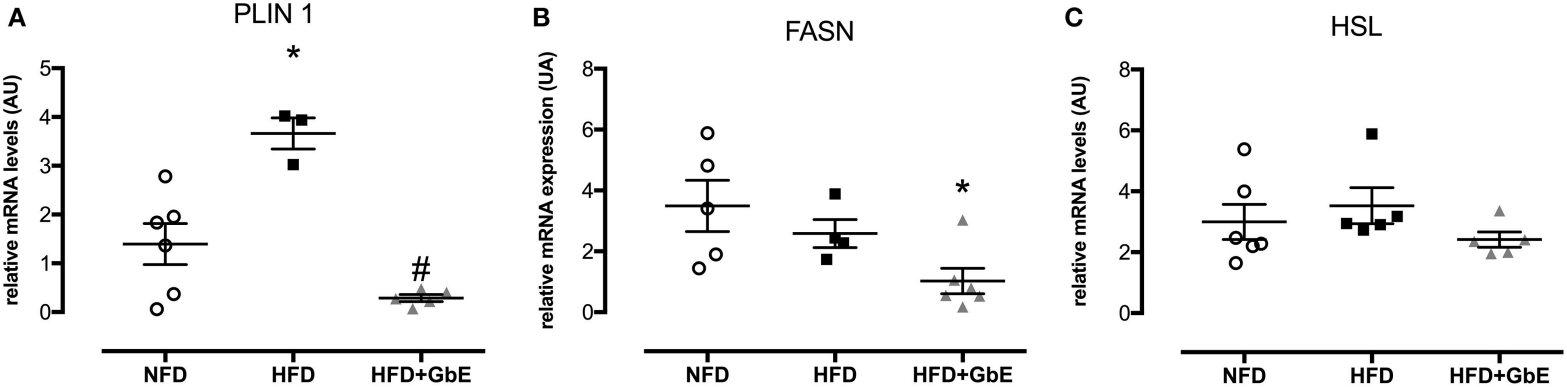
Gene expression. Effect of GbE supplementation on epididymal adipose tissue gene expression of PLIN1 **(A)**, FASN **(B)**, and HSL **(C)**, normal-fat diet (NFD; *n* = 6–4), high-fat diet (HFD; *n* = 5–3), and high-fat diet plus GbE supplementation (HFD+GbE; *n* = 6–4) groups. ^*^p < 0.05 vs. NFD and #*p* < 0.05 vs. HFD (One-way ANOVA). Gene expression in epididymal WAT depot was evaluated by Real Time PCR.

*PLIN*-1 (perilipin 1), *FASN* (fatty acid synthase), and *HSL* (hormone-sensitive lipase) epididymal adipose tissue mRNA expression were quantified by real time PCR. *PLIN* 1 gene expression (Figure 6A) was increased by 335% (*p* = 0.06) in HFD in comparison to NFD but decreased by 95.2% in HFD+GbE (*p* = 0.01) in comparison to HFD.

**Figure 7:**
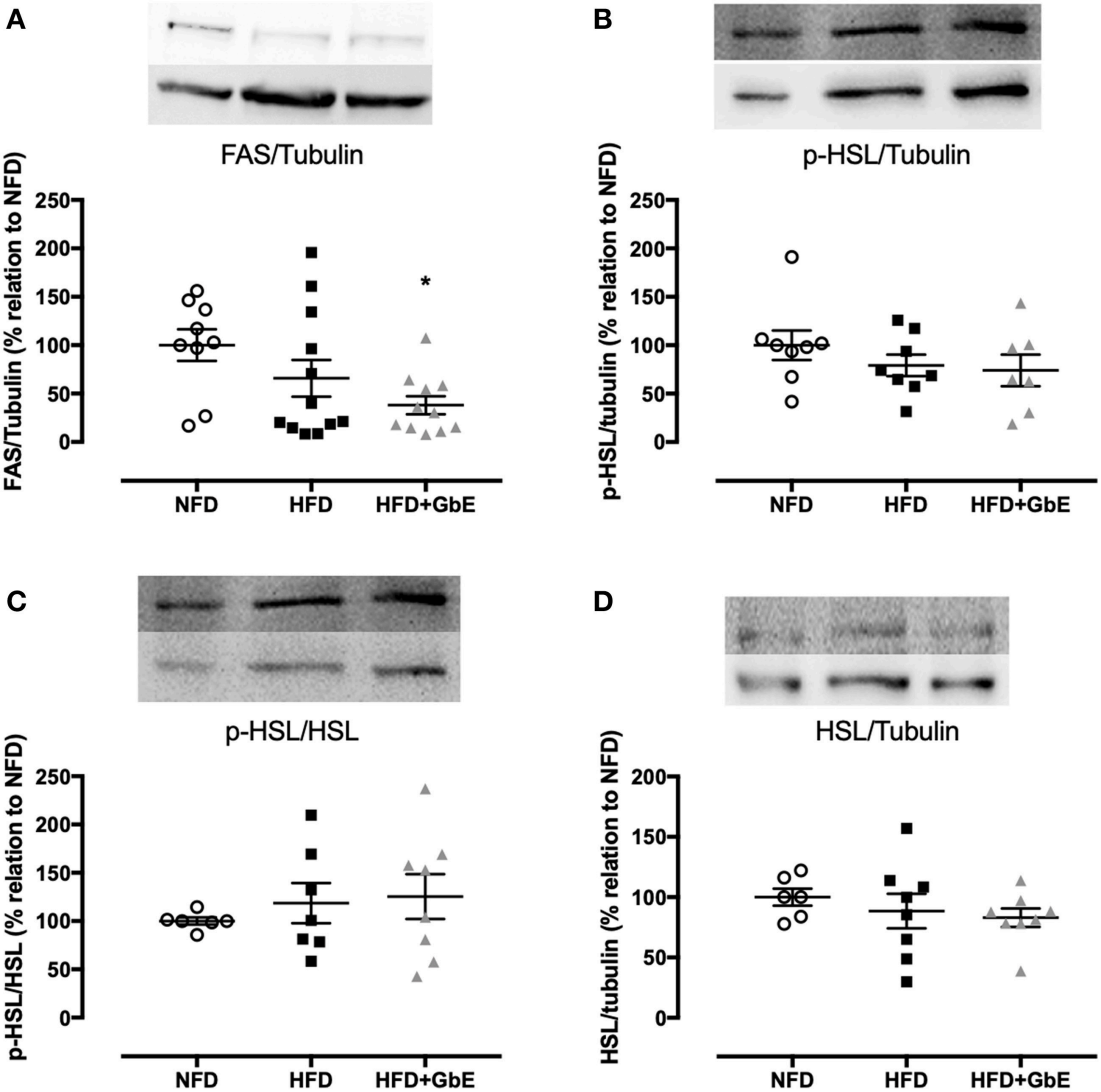
Epididymal protein expression. Protein synthesis of FAS **(A)**, p-HSL/tubulin **(B)**, p-HSL/HSL **(C)**, and HSL/tubulin **(D)** of normal fat diet (NFD; *n* = 9–6), high-fat diet (HFD; *n* = 12–7), and high-fat diet +GbE (HFD+GbE; *n* = 11–8). ^*^p < 0.05 vs. NFD (One-way ANOVA).

No statistically significant differences were found in *FASN* gene expression between HFD and NFD, but GbE supplementation significantly decreased it by 70.7% in relation to NFD (Figure 6B, *p* = 0.03). The *FASN* gene expression experiment was followed by tissue FAS protein quantification by western blotting. We have confirmed that there were no differences in FAS protein between NFD and HFD, but GbE supplementation did reduce tissue FAS content by 38% (*p* = 0.05) in relation to NFD (Figure 7A). No statistically significant differences were found in p-HSL and HSL protein amongst the three groups (Figures 7B–D).

## Discussion

GbE is the most widely herbal supplement therapy used worldwide (36). Its effects have been investigated in a range of chronic diseases including NAFLD (22, 23), Alzheimer's disease (37), memory loss (31), and cancer (38). Recent investigations from our research group have identified some of the molecular effects of GbE supplementation in obese rats. We have previously showed that GbE supplementation significantly reduced appetite and significantly stimulated hypothalamic serotonergic activity in obesity-associated ovariectomized rats (28). Additionally, diet-induced obese rats showed significant improvement in insulin sensitivity and increased insulin receptor (IR) and AKT phosphorylation after GbE supplementation (27). Collectively, these results suggest a potentially beneficial effect of GbE as coadjuvant for the treatment of obesity. In order to further test this hypothesis, in the current investigation we examined other markers of lipid metabolism on epididymal white adipose tissue of diet-induced obese rats supplemented with GbE.

In the present study, we observed a lower food intake (g/100 g/24 h) but higher relative energy intake (kcal/100 g/24 h) in HFD-fed rats throughout the 2 months of obesity-induction period, prior to GbE intervention. The HFD was effective in inducing obesity; HFD-fed rats showed significantly increased body weight in comparison to NFD-fed rats, as well as increased masses of retroperitoneal and epididymal adipose tissue. Such findings have been described previously, and it is well-known that lard-based high-fat diets increase body weight and visceral fat accumulation (26, 39, 40).

Further metabolic manifestations have been observed in lard-fed obese Wistar rats. Dornellas et al. (41) showed that the intake of lard-enriched high fat diet for 8 weeks resulted in significantly decreased linoleic acid, alpha-linolenic acid and the respective elongated desaturated omega 6 and 3 PUFAs in liver, serum, retroperitoneal, epididymal and mesenteric white adipose tissue lipid extracts. Due to the similarities between the experimental model used in our study and the one employed by Dornellas et al., it is reasonable to suggest that the content of essential fatty acids was decreased in our HFD rats, which may help to explain the phenotypic disturbances found in these animals.

We found at the end of the 2 weeks GbE supplementation period reduced food intake and reduced relative energy intake in GbE supplemented rats, in comparison to the non-supplemented obese ones. Similar results were observed in previous studies from our research group (26, 27). It has been proposed that *Ginkgo biloba* may stimulate serotoninergic systems, inducing hypophagia (28).

The reduced energy intake observed in HFD+GbE did not lead to significantly reduced body weight gain; both HFD and HFD+GbE showed statistically similar body weight at the end of the 2-week supplementation period. However, we have previously demonstrated that GbE induced a significant reduction in the mass of retroperitoneal and epididymal fat pads of obese rats (26). Furthermore, epididymal adipocytes harvested from HFD rats were significantly larger than those harvested from NFD rats, but the present data also showed that GbE supplementation induced a reduction in adipocyte volume of obese rats, bringing this index down to a volume similar to the NFD group.

This is, in our opinion, a remarkable effect of GbE supplementation. Adipocyte hypertrophy is a classical manifestation of obesity and is directly associated with the aetiology of macrophage infiltration, hypoxia, secretion of pro-inflammatory cytokines (e.g., TNF-α, IL-6, IL-1β) and reduced production of adiponectin. Such disturbances induce low grade inflammation and insulin resistance (11, 42). Not only that, abnormally expanded adipocytes have a reduced capacity to further store additional amounts of triacylglycerol, leading to its ectopic deposition in other organs such as skeletal muscle, heart, pancreas, and liver (3, 43). A reduction of adipocyte volume without concomitant losses in body weight, which we did find in the present study, could well possibly suggest an improvement in body composition. Further studies are however needed to further investigate this hypothesis.

The reduced adipocyte volume observed in the HFD+GbE rats may be associated with their lowered food intake; however, we do not believe these findings suggest starvation. Calorie restriction has been associated with starvation signals such as reduced leptin and insulin (44), but in the opposite direction, we have previously found increased insulin levels and activity in obese rats supplemented with GbE submitted to the same experimental protocol (26).

Increased visceral adiposity and perturbations in adipocyte volume are only a few of the manifestations seen in adipose tissue in obesity; lipid uptake and lipolysis rate are also disturbed (45). In the present study, the lipolysis index was quantified by the amount of glycerol released from incubated adipocytes into the incubation medium. Isoproterenol, a sympathomimetic compound, was added to mimic adrenergic activation upon lipolysis. We found no statistically significant differences in either basal or isoproterenol-stimulated lipolysis amongst the three groups investigated. It is known however that lipolysis rate is higher in obesity (46). Corroborating this observation, in the current investigation, despite the absence of statistically significant differences in lipolysis rate amongst the three groups, HFD+GbE adipocytes showed basal lipolysis rate ~45% lower, and isoproterenol-stimulated lipolysis ~15% lower than HFD adipocytes.

We also quantified in *ex-vivo* epididymal adipocytes the fatty acid incorporation into TAG, defined as intracellular accumulation of [3^H^]-oleate. Our results show that incorporation was significantly higher in HFD in comparison to NFD (Figure 5E), and GbE reduced it by 43% in relation to HFD (Figure 5E).

In the present investigation, lipolysis rate remained statistically similar between HFD and NFD. Furthermore, oleate incorporation was significantly higher in HFD. Taken these results together, it is expected that in the longer term HFD adipocytes would only expand their volumes further, deteriorating the molecular consequences of obesity. However, our results suggest that GbE would be effective in impairing such deterioration, as lipolysis rate and fatty acid incorporation were lower in the rats that received it as compared to the ones that did not. Both biomarkers were also statistically similar between HFD+GbE and NFD. Our hypothesis is further corroborated by the findings of intracellular incorporation of [1-^14^C]-acetate, presented in Figure 6E. Acetate is a key component in fatty acid synthesis, and we found significantly reduced acetate incorporation into lipids in the HFD+GbE group in relation to HFD.

*Ginkgo biloba* extract is a rich mixture of polyphenolic compounds, including ginkgolides, bilobalides, quercetin, and several others (47). Previous studies showed that 3T3-L1 adipocytes incubated with ginkgolide C showed increased ATGL and HSL activity and AMPK phosphorylation, as well as decreased activity of acetyl-CoA carboxylase for fatty acid synthesis (30). Furthermore, 3T3-L1 cells subjected to hypoxia and incubated with bilobalide showed reduced pro-inflammatory biomarkers and improved insulin sensitivity (48). Similarly, ginkgetin, a biflavone from *Ginkgo biloba*, has been described as a STAT5 inhibitor, blocking the differentiation of pre-adipocytes into adipocytes harvested from high-fat diet-induced obese mice (49). Moreover, OP9 cells, which differentiate into adipocytes *in vitro*, showed reduced adipogenesis, increased HSL and decreased FAS expression upon treatment with quercetin (50). Such results confirm previously published findings that 3T3-L1 adipocytes treated with a range of biflavones found in *Ginkgo biloba* showed increased lipolysis rate (29), and that biflavones inhibit cAMP-phosphodiesterase in rat adipose tissue (51).

Perilipin 1 is an intracellular protein that covers the lipid droplet surface in adipocytes, regulating TAG storage and mediating stimulated lipolysis (13). Higher perilipin activity (52) and higher TNF-α levels (53) are observed in obesity. TNF-α induces lipolysis mediated by the activation of perilipin 1, promoting its displacement from the lipid droplet, making the TAG molecule more accessible to HSL (43).

In the present investigation, we found lower perilipin 1 relative mRNA levels in HFD+GbE as compared to HFD, down to levels similar to the NFD group. The lower perilipin levels found in GbE-supplemented rats may suggest that a lower lipolysis rate will occur in the longer term, further corroborating the lipolysis findings described above. Also, it further supports the hypothesis that GbE supplementation may lead to lower fat accumulation in the long term.

Fatty Acid Synthase (FAS) is another pivotal protein in the regulation of body adiposity, utilizing malonyl-CoA and acetyl-CoA for the synthesis and elongation of fatty acid chains, which are eventually incorporated into lipid droplets (13). Higher levels of *Fasn* mRNA and FAS protein have been described in obesity, in visceral fat accumulation, and in enlarged insulin-resistant adipocytes (54–56). In the present study, we found decreased *Fasn* mRNA gene expression and FAS protein synthesis in the HFD+GbE group in comparison to the NFD group, which suggests GbE could have a FAS-inhibiting effect.

FAS has been proposed as a potential therapeutic target for the treatment of obesity (54, 57), and grape skin extract and resveratrol have shown FAS enzyme-inhibiting properties. In 3T3-L1 pre-adipocytes, resveratrol reduced lipid accumulation (58). Furthermore, resveratrol treatment decreased the epididymal adipose tissue *Fasn* mRNA expression in high fat diet-fed mice (59). FAS inhibition is followed by malonyl-CoA, acetyl-CoA and NADPH accumulation, which is interpreted by the cell as a signal for abundance of energy (58).

Moreover, FAS may be an important modulator of feeding regulation. C75 is a synthetic inhibitor of FAS found to suppress food intake in obese mice mediated by NPY/AgRP orexigenic neurons, also increasing the expression of Melanin-Concentrating Hormone and its receptor in the hypothalamus. C75 reduced body weight and body fat content, and normalized obesity-associated hyperglycaemia and hyperinsulinemia (55, 57, 60). In the present study, we observed lower food and calorie intake in the HFD+GbE rats, which may be associated with decreased levels of FAS gene expression and protein synthesis.

In summary, our study shows that rats fed a high fat diet for 2 months and subsequently supplemented with GbE for 2 weeks showed significant reduction in several biomarkers of lipid metabolism, including reduced epididymal adipocyte volume and [1-^14^C]-acetate incorporation into fatty acids, *Plin 1* mRNA, *Fasn* mRNA, and protein levels, alongside a tendency for reduced epididymal [^3^H]-oleate incorporation. Furthermore, the energy intake of GbE-supplemented rats was significantly lower than of rats receiving a control diet and of rats received the high fat diet only.

These findings allow us to suggest that GbE supplementation might be a promising alternative anti-obesogenic therapeutic approach. This is of particular relevance for obese patients who do not successfully engage in nutritional and positive lifestyle re-education programmes. However, due to the limitations of this study, as it involves a rodent model, further studies are necessary to test the validity of our hypothesis.

## Ethics Statement

This study was carried out in strict accordance with the recommendations of the Guide for the Care and Use of Laboratory Animals. The Committee on Animal Research Ethics of the Universidade Federal de São Paulo approved all procedures for the care of the animals used in this study (Process number: 8700110814).

## Author Contributions

BH contributed to conception and design of the work, acquisition, analysis and interpretation of data for the work, drafted and revised the manuscript and provided final approval of the submitted version, responsible for all study aspects including its accuracy and integrity. MC, RdS, TF, and MM contributed to the data acquisition, analysis and interpretation, revised the manuscript and provided final approval of the submitted version, responsible for all study aspects including its accuracy and integrity. AB contributed to the data analysis and interpretation; drafted and revised the manuscript, revised the manuscript and provided final approval of the submitted version, responsible for all study aspects including its accuracy and integrity. MA-V and MT contributed to conception and design of the work, analysis and interpretation of data for the work, drafted and revised the manuscript and provided final approval of the submitted version, responsible for all study aspects including its accuracy and integrity. All authors approved the final version of the manuscript.

### Conflict of Interest Statement

The authors declare that the research was conducted in the absence of any commercial or financial relationships that could be construed as a potential conflict of interest.
