# Supplementary material for: Potential Anti-obesogenic Effects of Ginkgo biloba Observed in Epididymal White Adipose Tissue of Obese Rats
Source: Front Endocrinol (Lausanne). 2019 May 10;10:284. doi: 10.3389/fendo.2019.00284 (PMC6523993; doi:10.3389/fendo.2019.00284)
Supplement: Supplementary file 1 [file Data_Sheet_1.DOCX]

**FAS**

**
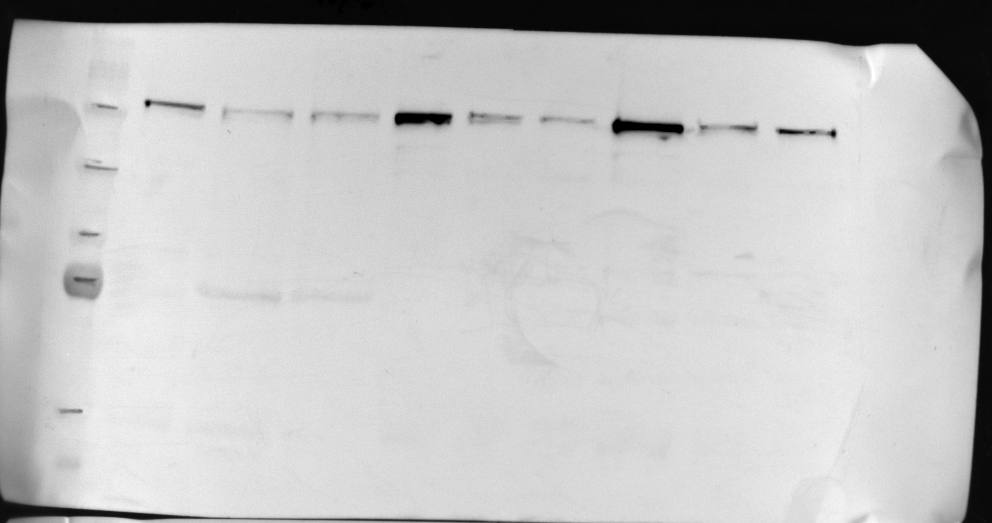
**

**NFD HFD HFD+GbE NFD HFD HFD+GbE NFD HFD HFD+GbE**

FAS

150kDa

250kDa

**
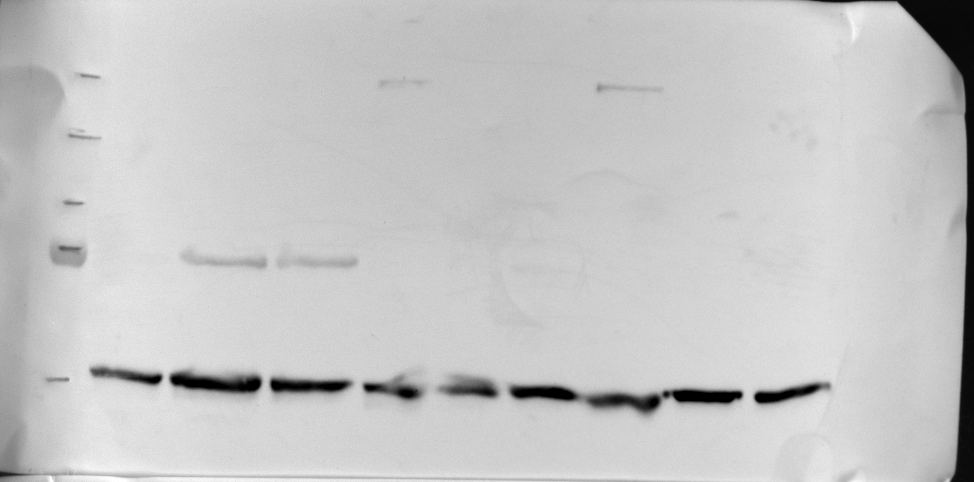
**

**NFD HFD HFD+GbE NFD HFD HFD+GbE NFD HFD HFD+GbE**

Beta-Tubulin

75kDa

50kDa

**p-HSL**

**NFD HFD HFD+GbE NFD HFD HFD+GbE NFD HFD HFD+GbE**

**
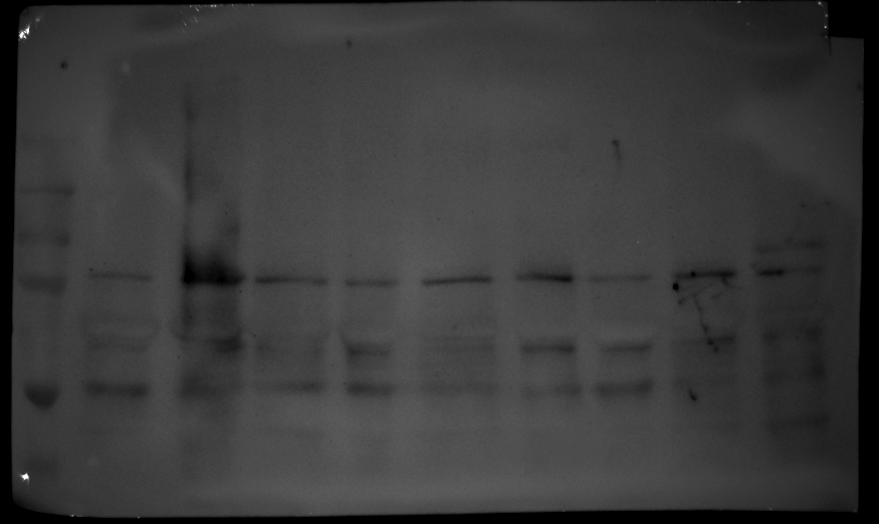
**

50kDa

p-HSL

75kDa

***
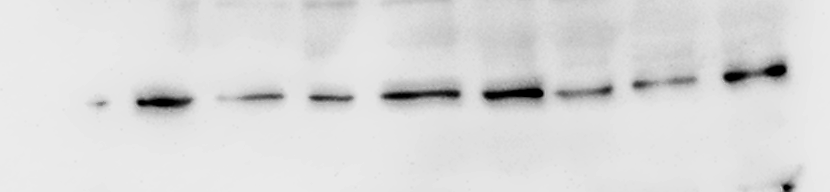
***

**NFD HFD HFD+GbE NFD HFD HFD+GbE NFD HFD HFD+GbE**

Beta-Tubulin

50kDa

**HSL**

**
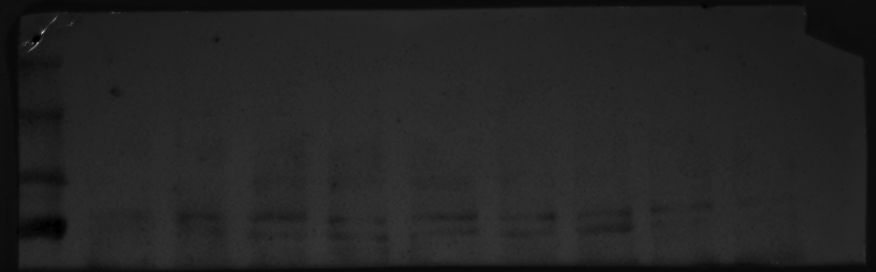
**

100kDa

HSL

75kDa

**NFD HFD HFD+GbE NFD HFD HFD+GbE NFD HFD HFD+GbE**

**
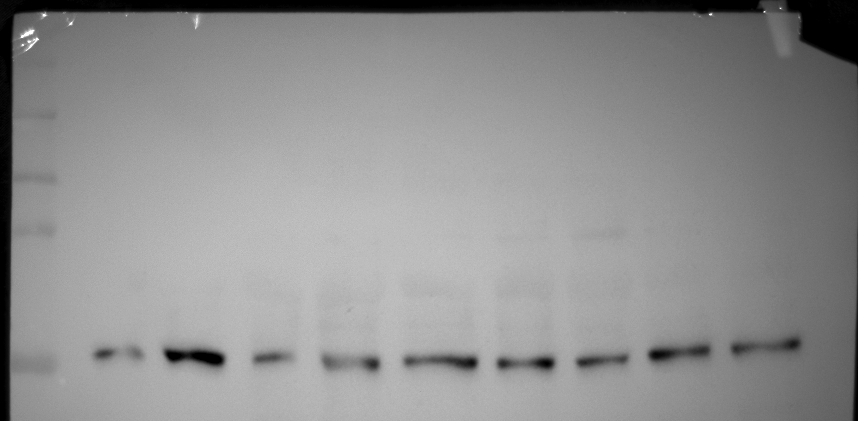
**

Beta tubulin

50kDa

**NFD HFD HFD+GbE NFD HFD HFD+GbE NFD HFD HFD+GbE**
